# Laboratory Electrical Resistivity Studies on Cement Stabilized Soil

**DOI:** 10.1155/2017/8970153

**Published:** 2017-04-30

**Authors:** Nimi Ann Vincent, R. Shivashankar, K. N. Lokesh, Jinu Mary Jacob

**Affiliations:** ^1^Civil Engineering Department, National Institute of Technology, Karnataka, Surathkal, India; ^2^Larsen and Toubro Constructions, Chennai, India

## Abstract

Electrical resistivity measurement of freshly prepared uncured and cured soil-cement materials is done and the correlations between the factors controlling the performance of soil-cement and electrical resistivity are discussed in this paper. Conventional quality control of soil-cement quite often involves wastage of a lot of material, if it does not meet the strength criteria. In this study, it is observed that, in soil-cement, resistivity follows a similar trend as unconfined compressive strength, with increase in cement content and time of curing. Quantitative relations developed for predicting 7-day strength of soil-cement mix, using resistivity of the soil-cement samples at freshly prepared state, after 1-hour curing help to decide whether the soil-cement mix meets the desired strength and performance criteria. This offers the option of the soil-cement mix to be upgraded (possibly with additional cement) in its fresh state itself, if it does not fulfil the performance criteria, rather than wasting the material after hardening.

## 1. Introduction

Soil electrical resistivity testing has been gaining importance in geotechnical and geoenvironmental fields due to its time efficiency and cost. Soil electrical resistivity testing will be cheaper and faster than conventional laboratory testing when dealing with a large number of soil samples. The electrical measurement method is one of the nondestructive methods which can be applied both in the laboratory and in the field and considered as nondestructive geophysical method in the case of the latter. Electrical resistivity of soils depends on many factors such as porosity, nature of ions present in the pore fluid, mineral composition of the solids, density, degree of saturation, particle shape, orientation, and pore structure. The fundamental principle of the soil investigation with electrical resistivity is that when a constant voltage is applied to one of the two probes placed in the soil, the current that flows between the probes is inversely relative to the resistance of the soil. Electrical resistivity shows indeed strong variations that principally depend on soil water content variations.

Bery and Saad [1] performed laboratory electrical resistivity tests for the engineering characterization of a clayey sand soil. The empirical correlations between electrical parameter, liquid limit, plastic limit, plasticity index, moisture content, internal friction angle, and effective soil cohesion were obtained. The results showed that internal friction angle is inversely proportional to the resistivity of samples and effective cohesion is directly proportional to the resistivity.

Stabilization of soils with additives helps to increase strength, reduce deformability, provide volume stability, reduce permeability, and reduce erodibility. Wei et al. [2] established a linear relationship between 28-day compressive strength and resistivity of cement paste after 24 hours. The compressive strength of cement paste at 28 days could be predicted easily using a quantitative relation developed with resistivity of cement paste at 24 hours. In this study, electrical resistivity measurements of freshly prepared uncured and cured soil-cement materials are carried out and the correlation between the factors controlling the performance of soil-cement and electrical resistivity are being studied. Simple regression equations are developed between electrical resistivity and strength parameters in an attempt to predict the performance of soil-cement mixtures with respect to compressive, tensile, and the shear strength parameters in the fresh state itself without having to wait for 7 days. Moreover, predicting the strength parameters at the freshly prepared state could be advantageous and a necessity in many cases. If the soil-cement material does not meet the strength requirement as per the resistivity models developed, additional cement constituents could be added in the fresh state itself and used. This will prevent wasting of the materials after hardening. The additional advantages of the technique are that it is quick and nondestructive.

## 2. Electrical Resistivity of Stabilized Soils

Liu et al. [3] conducted a study for investigating the factors controlling the electrical resistivity of soil-cement admixtures.

### 2.1. Effect of Cement Content

With the increase in cement content, water content and void ratio of the soil-cement admixture get decreased due to the hydration reaction and pozzolanic reaction. As a result, the path for the conduction of electrical current becomes more tortuous. Therefore, the electrical resistivity of the soil-cement admixture increased. The higher the cement content, the higher the hydration compounds formed.

### 2.2. Effect of Degree of Saturation

Electrical resistivity increased with the decrease in degree of saturation because less pore spaces were filled with pore water, as water gets utilized for hydration reaction and thus continuous water bridging is not available for electrical conduction.

### 2.3. Effect of Water Content on Electrical Resistivity

With decrease in water content, the tortuosity of the conduction path for the electrical current increases, resulting in increase of electrical resistivity.

### 2.4. Effect of Curing Time on Electrical Resistivity

With the increase in the curing time, the chemical reaction products such as calcium silicate hydrate (CSH) and calcium aluminate hydrate (CAH) formed bind finer soil particles together resulting in a denser soil structure. Hence electrical resistivity is increased.

Zhang et al. [4] worked on quantifying the effect of cement content, porosity, and curing period on the electrical resistivity and UCS of cement treated soil. The general Archie law, which includes the effect of water content and porosity, was modified to evaluate the effect of cement content and curing periods on the electrical resistivity of cement stabilized soil. Archie [5] developed an empirical relationship that relates the electrical resistivity of saturated sand (*ρ*) to the electrical resistivity of its pore fluid (*ρ*_*w*_) and the porosity (*n*) of the soil.

(1) $$\begin{array}{c} \frac{\rho }{\rho_{w}}=n^{-m}, \end{array}$$

where *m* is the material-dependent empirical exponent, which is a measure of pore tortuosity and the interconnectivity of the pore network.

A new parameter, termed as (after curing porosity/cement content-curing time) ratio, *n*_*t*_/(*a*_*w*_*T*), was proposed to relate the electrical resistivity values as

(2) $$\begin{array}{c} \rho =A{\left(\;\frac{n_{t}}{a_{w}T}\;\right)}^{-B}, \end{array}$$

where *A* and *B* are dimensionless constants.

## 3. Materials Used

**Figure 1:**
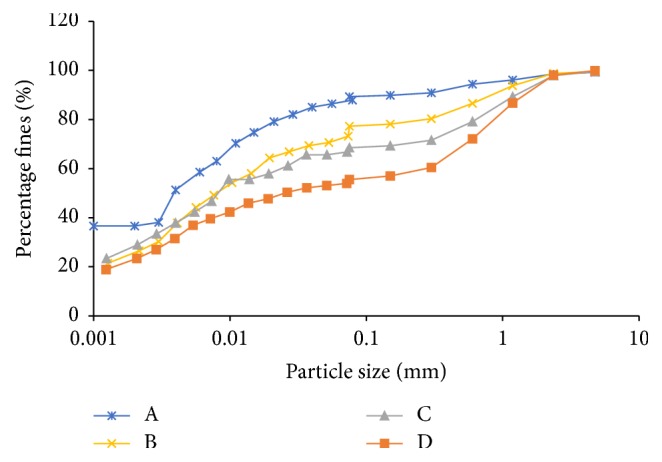
Sieve analysis results of soil samples.

The soils used in the present study are lithomargic soils, which are products of laterization. These soils are locally called “Shedi soils” and are available in varied colours. These soils are characterized by high silt content and low strengths [6]. In order to vary the percentage of fines, in the different test samples, controlled soil samples were prepared. River sand was used for blending the Shedi soil. All these soil samples were used to study the geotechnical and electrical properties. The percentages of river sand used were 0, 10, 20, and 30% by weight of dry soil. The samples are designated as A, B, C, and D, respectively. For the experimental investigations, river sand passing IS 4.75 mm sieve and retained on IS 75-micron sieve was considered. The sieve analysis curves of the samples are shown in Figure 1.

## 4. Cement

43-grade Ordinary Portland Cement (OPC) was used in the study. The percentages of cement used were 2%, 4%, and 6% in each of the four soil samples, A, B, C, and D. These samples are designated as A2, A4, A6, B2, B4, B6, C2, C4, C6, D2, D4, and D6, respectively.

## 5. Test Method

**Figure 2:**
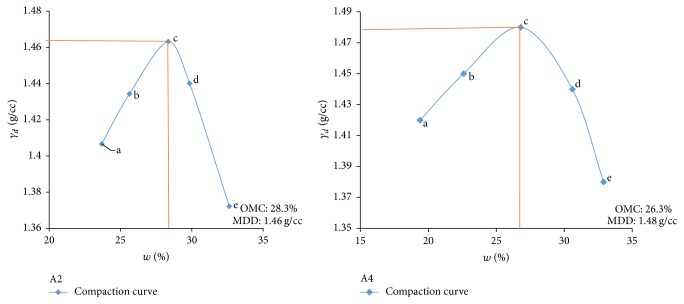
Light compaction curve for samples A2 and A4.

**Figure 3:**
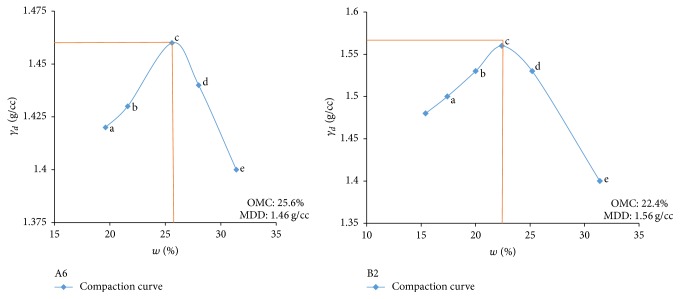
Light compaction curve for samples A6 and B2.

**Figure 4:**
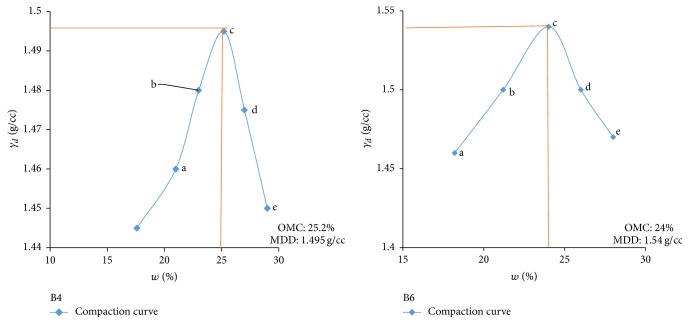
Light compaction curve for samples B4 and B6.

**Figure 5:**
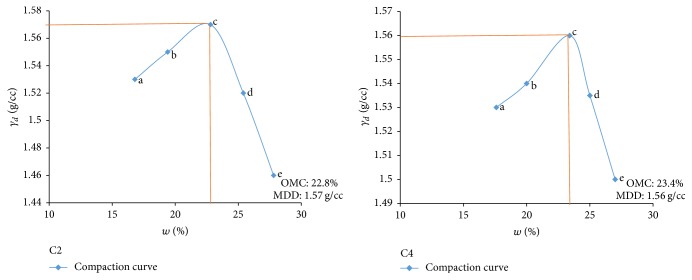
Light compaction curve for samples C2 and C4.

**Figure 6:**
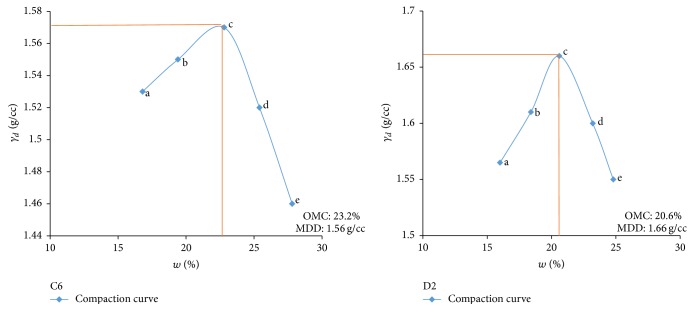
Light compaction curve for samples C6 and D2.

**Figure 7:**
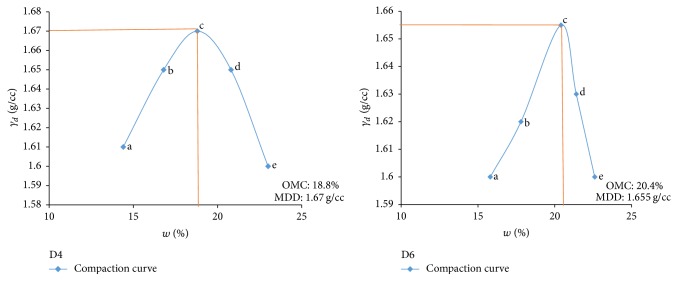
Light compaction curve for samples D4 and D6.

**Figure 8:**
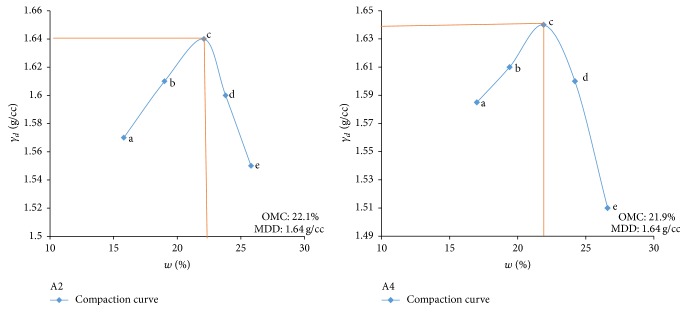
Heavy compaction curve for samples A2 and A4.

**Figure 9:**
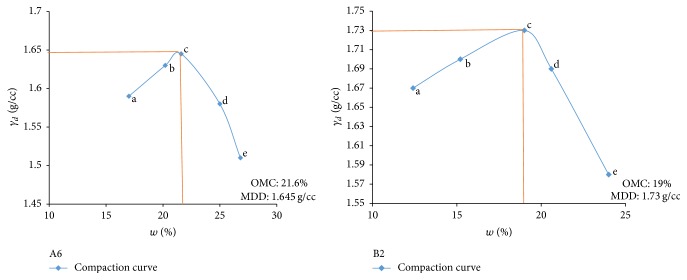
Heavy compaction curve for samples A6 and B2.

**Figure 10:**
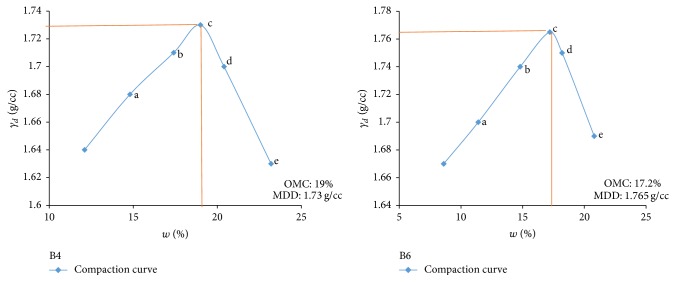
Heavy compaction curve for samples B4 and B6.

**Figure 11:**
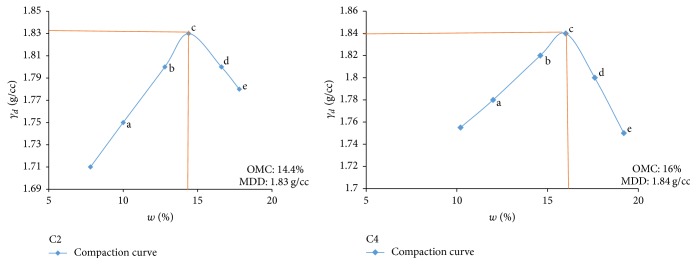
Heavy compaction curve for samples C2 and C4.

**Figure 12:**
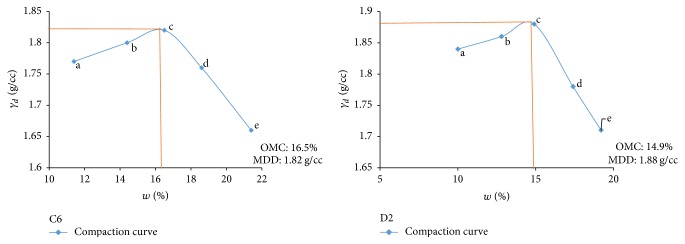
Heavy compaction curve for samples C6 and D2.

**Figure 13:**
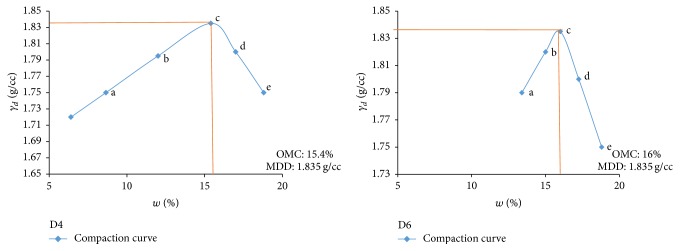
Heavy compaction curve for samples D4 and D6.

Electrical resistivity of all the controlled samples was done by making cylindrical samples of size 7.6 cm height and 3.8 cm diameter. For each combination, in addition to the point of maximum dry density and optimum moisture content obtained from Standard Proctor and Modified Proctor tests, two points each were selected on the dry side and wet side of the compaction curve to study resistivity variation for different compaction conditions (Figures 2–13). Figures 2–7 show the compaction curve for light compaction (standard Proctor compaction) and Figures 8–13 show compaction curve for heavy compaction (modified Proctor compaction).

**Figure 14:**
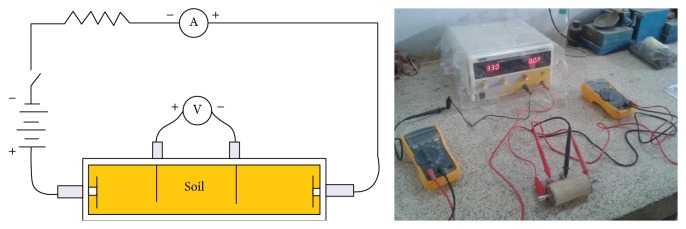
Resistivity measurement.

The resistivity measurements were taken for all the seven curing days. Electrical resistivity was measured by using a circuit consisting of a 30 V DC power supply, two high precision multimeters serving as ammeter and voltmeter, and electrodes connecting to the sample as seen in Figure 14. Two circular steel plates are placed touching the two ends of the sample which acts as current electrodes and two steel pins at one-third length from both ends act as voltage electrodes. The stainless steel electrodes were arranged in Wenner *α* configuration. Wenner *α* array is less affected by the electrode position error compared to dipole-dipole array [7].

Resistivity was measured in the freshly prepared state, after one-hour curing and after one to seven days of curing. The basic geotechnical properties of the soil stabilized with different percentages of cement are given in Table 1.

## 6. Results and Discussions

Compaction and strength characteristics of the soil-cement samples are shown in Table 1.

### 6.1. Variation of Electrical Resistivity with Time of Curing

**Figure 15:**
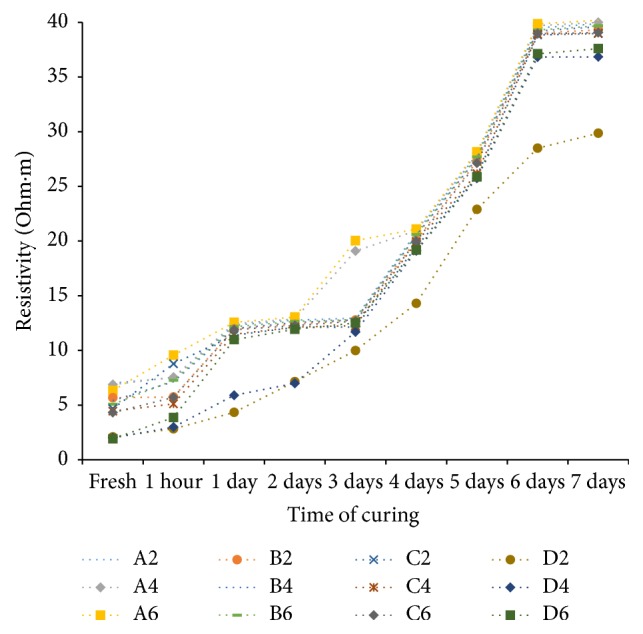
Variation of resistivity at OMC and corresponding maximum dry density with time of curing.

From Figure 15, which shows variation of resistivity at OMC and corresponding maximum dry density with time of curing, it is seen that resistivity increases with curing period. The resistivity results show that, for all the soil-cement samples, at freshly prepared state and after one hour of curing time, resistivities are high when compacted on dry side of optimum. With increase of moisture content and dry density, resistivity decreases significantly. In the wet side, soil resistivity is low. The moulding water content which was available for electrical conduction at freshly prepared state gets utilized for hydration of cement, which depletes the free water film available for conduction, with time of curing.

### 6.2. Resistivity with Compaction Effort

**Figure 16:**
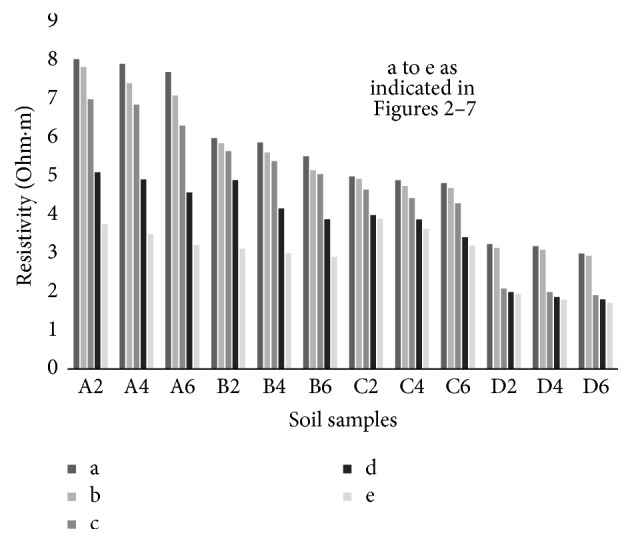
Variation of resistivity at dry side and wet side points on the standard Proctor compaction curve at day zero.

**Figure 17:**
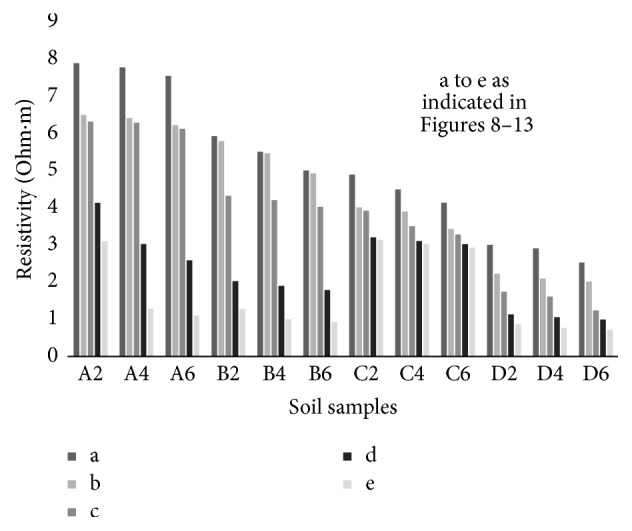
Variation of resistivity at dry side and wet side points on the modified Proctor compaction curve at day zero.

The electrical response of soil when the soil samples are compacted with different degree of compaction is also looked into. Light and heavy mechanical compaction were performed on the soil samples and the resistivity variation on these samples at different compaction condition is being studied. In the bar graphs (Figures 16 and 17), “a” and “b” represent the dry side of optimum compaction points. The point “c” represents the maximum compaction condition. The points “d” and “e” represent the wet side of compaction points (as shown in Figures 2–13). It is seen from Figures 16 and 17 that resistivity decreases with increase in water content and dry density on the dry side but is dependent only on water content in the wet side of the compaction curve.

**Figure 18:**
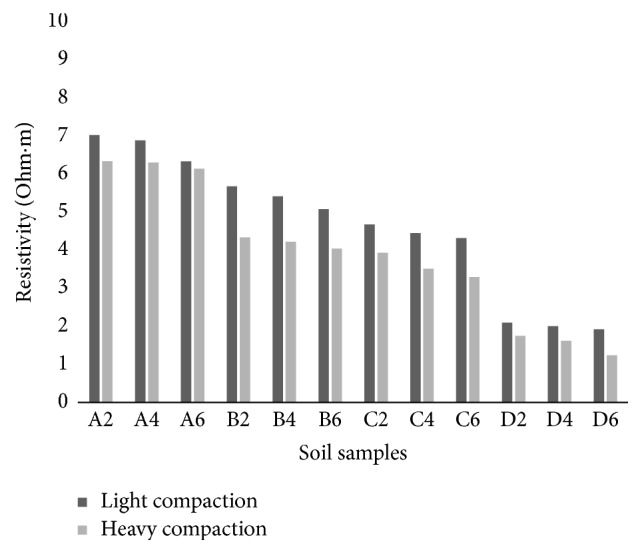
Variation of electrical resistivity at light (LC) and heavy (HC) day zero compaction conditions at OMC and maximum dry density conditions.

The solid particles of noncohesive soils are poor conductive while electrical current flow occurs only in intergranular spaces filled with mineralized water. As a consequence, the electrical conductivity of rocks and soils is clearly dependent on the amount of water in the medium, the conductivity of water, and how the water is spread (porosity, the degree of saturation, cementation factor, and fracturing). From Figure 18, it is observed that the resistivities are comparatively lower for the samples compacted at heavy compaction conditions than those compacted at light compaction conditions for all curing periods. At heavy compaction conditions, soil attains a denser state with higher degree of saturation and lesser air voids, which results in a lower apparent resistivity of the soils. At freshly prepared state and after one hour of curing, the ions present in the saturated and continuous micropores slightly exhibit higher electrical conduction and hence a lower resistivity in heavily compacted soil-cement samples compared to lightly compacted samples.

After 7 days of curing, the soil-cement samples harden and less water and ions will be available for conduction. The lightly compacted samples are more porous than heavily compacted dense samples. After 7 days of curing, water in these pores is utilized for hydration of cement and is replaced with air which offers infinite electrical resistance. Hence, after a period of seven days of curing the lightly compacted soil-cement samples exhibit higher resistivity than the heavily compacted ones.

### 6.3. Variation of Resistivity with Cement Content

**Figure 19:**
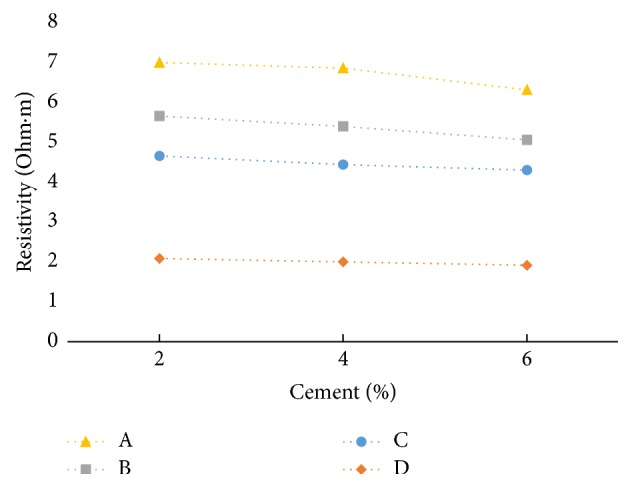
Resistivity (at freshly prepared state) versus cement content at LC (at max dry density and OMC).

**Figure 20:**
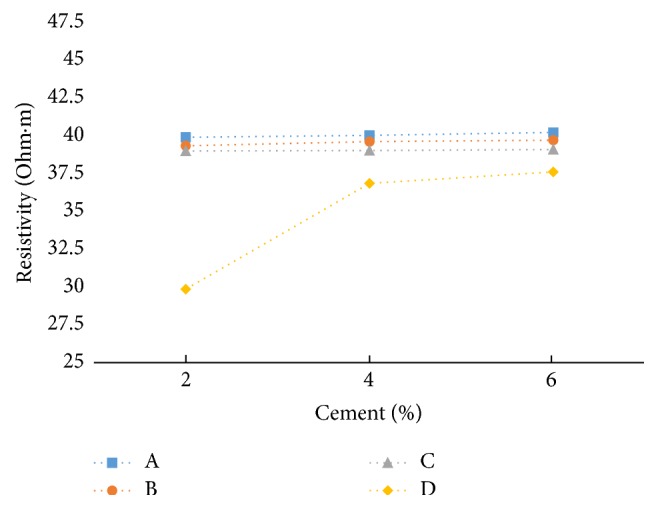
Resistivity (after 7 days' curing) versus cement content at LC (at max dry density and OMC).

It is observed that, in freshly prepared state, resistivity decreases slightly with cement content for all the soil-cement samples (Figure 19). But on the other hand, resistivity is slightly increasing with cement content when measured after curing (Figure 20).

Cement reduces the plasticity and water-retention capacity of the soil and improves its strength. Immediately after mixing, calcium (Ca) and hydroxyl (OH) ions go into solution. Then after a few minutes a slow precipitation of semicrystalline calcium silicate hydrate (CSH) gel occurs while the Ca and OH ions concentrations continue to increase slowly. Hence, initially the freshly prepared soil-cement samples show some conductivity, which diminishes with time. The Ca ion concentration reaches the saturation level, and the hydration reactions begin, with the crystallization of solid calcium hydroxide and the deposition of CSH gel in voids. While the structure is progressing up, the pore spaces decrease and the availability of ions and water will be lesser, which results in a higher electrical resistivity. The hydration compounds fill in pore spaces and intersect with each other to form a denser structure. In the meantime, the free water space and porosity decrease and tortuosity increases. Consequently, electrical resistivity increases more significantly [4].

### 6.4. Resistivity with Porosity

Porosity of all the sample combinations was found for all the curing days. Cement content has a great effect on electrical resistivity of soil-cement. The measured electrical resistivity of cement treated soils increases with the increase of cement content [4]. For a given curing time, higher cement content yields higher amount of hydration products resulting in a denser structure. With this, free water space and porosity decrease and tortuosity increases resulting in increase of electrical resistivity.

**Figure 21:**
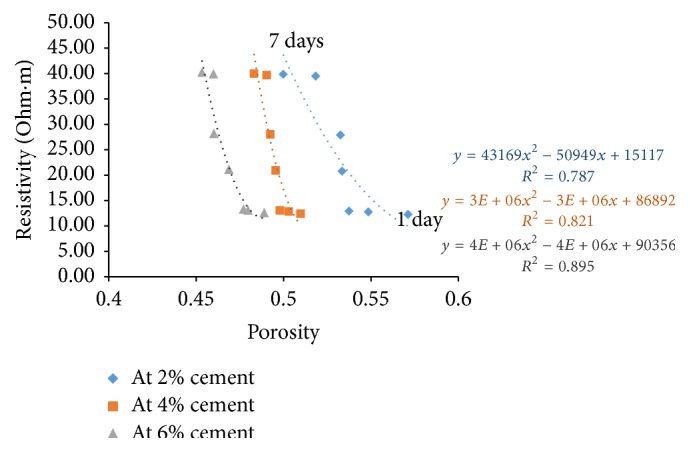
Variation of resistivity with porosity for sample A.

**Figure 22:**
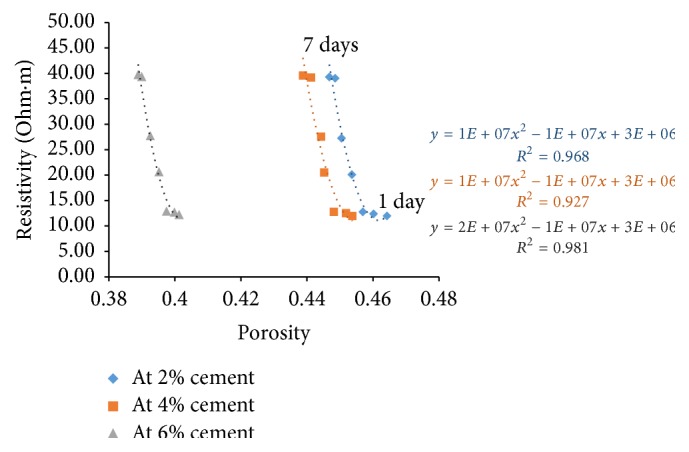
Variation of resistivity with porosity for sample B.

**Figure 23:**
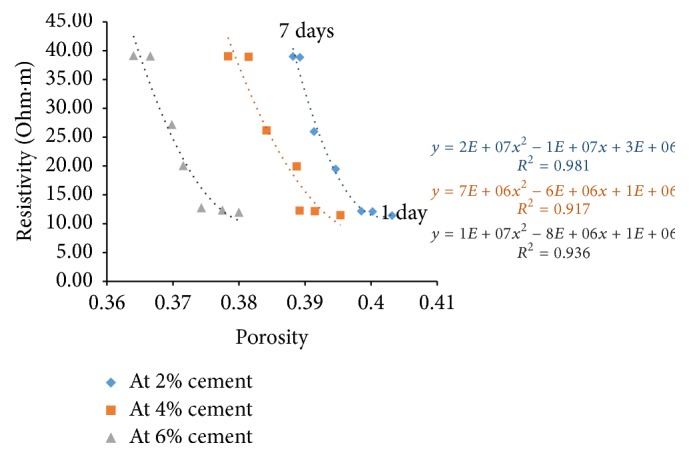
Variation of resistivity with porosity for sample C.

**Figure 24:**
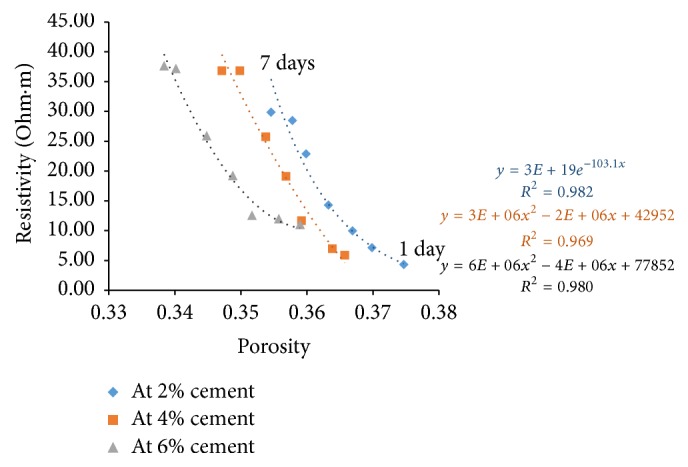
Variation of resistivity with porosity for sample D.

Figures 21–24 show the variation of electrical resistivity with porosity for varying percentages of river sand. It can be observed that, as cement content increases, porosity decreases. With curing time, for each percentage of cement, porosity decreases and the denser structure results in increase of resistivity.

### 6.5. Scanning Electron Microscope (SEM) Analysis

**Figure 25:**
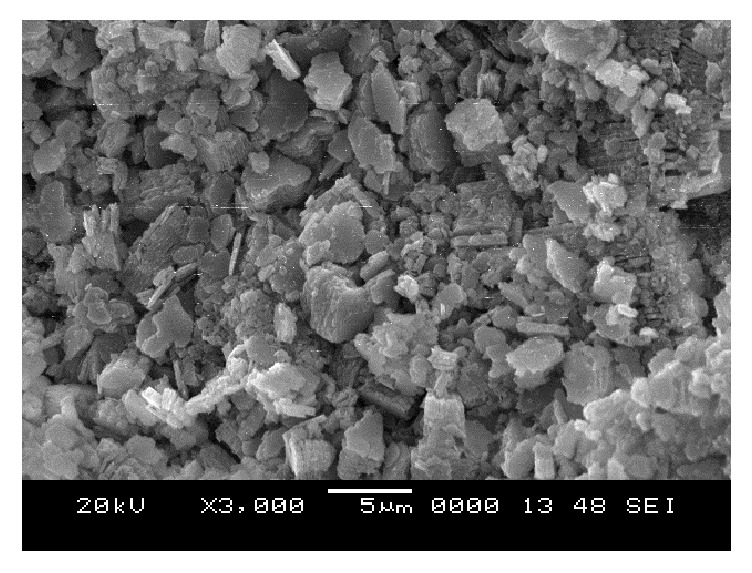
SEM image of soil-cement mix A2, at freshly prepared state.

**Figure 26:**
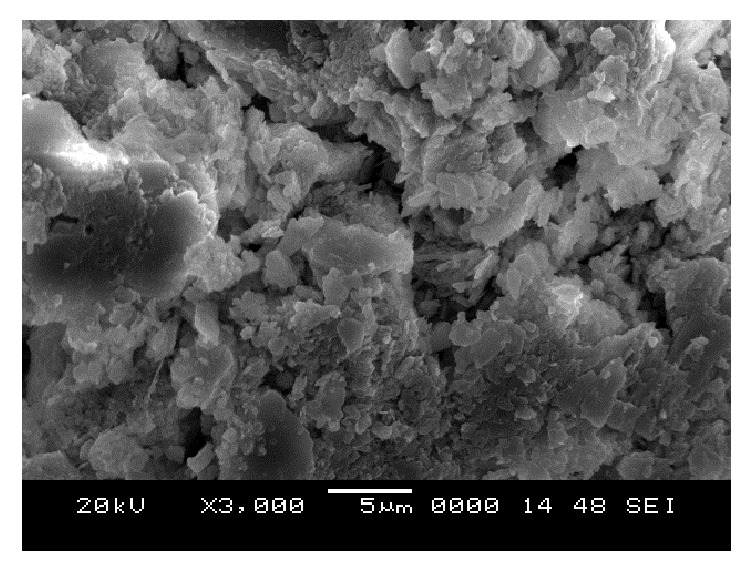
SEM image of soil-cement mix A2, after 1-day curing.

**Figure 27:**
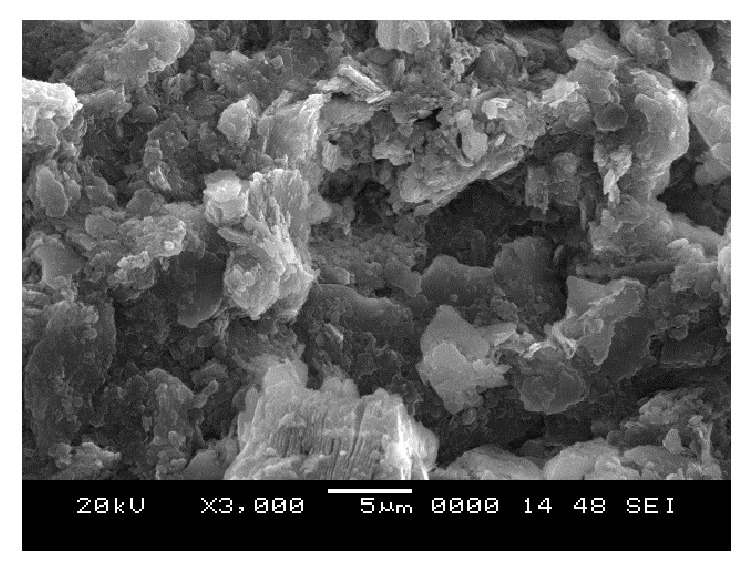
SEM image of soil-cement mix A2, after 2 days' curing.

**Figure 28:**
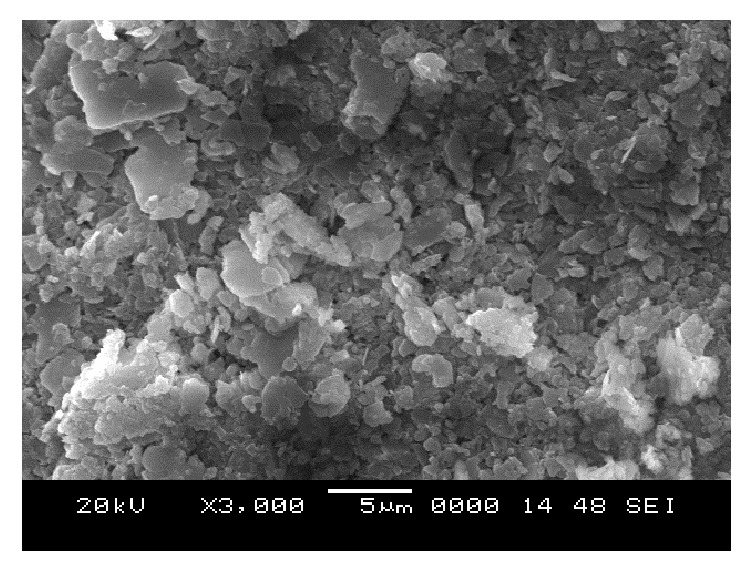
SEM image of soil-cement mix A2, after 3 days' curing.

**Figure 29:**
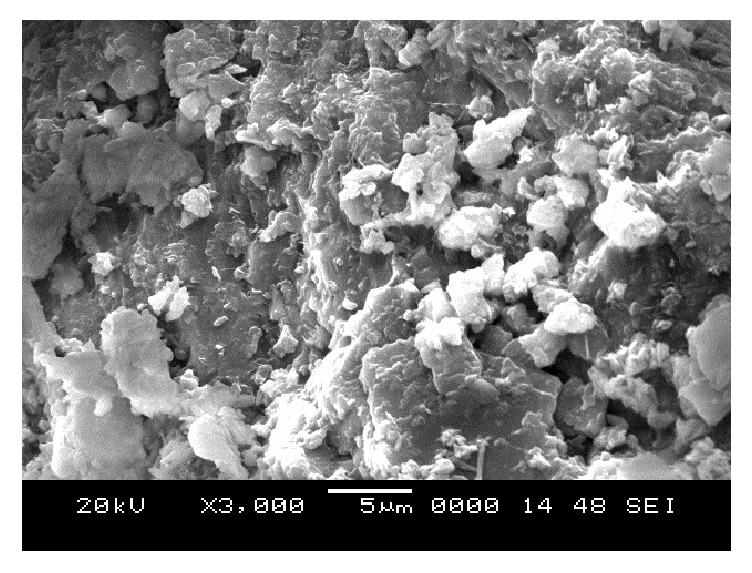
SEM image of soil-cement mix A2, after 4 days' curing.

**Figure 30:**
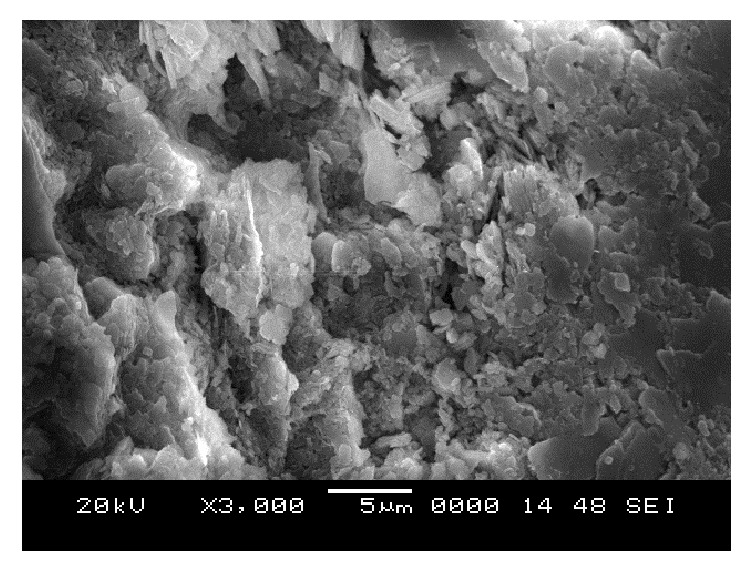
SEM image of soil-cement mix A2, after 5 days' curing.

**Figure 31:**
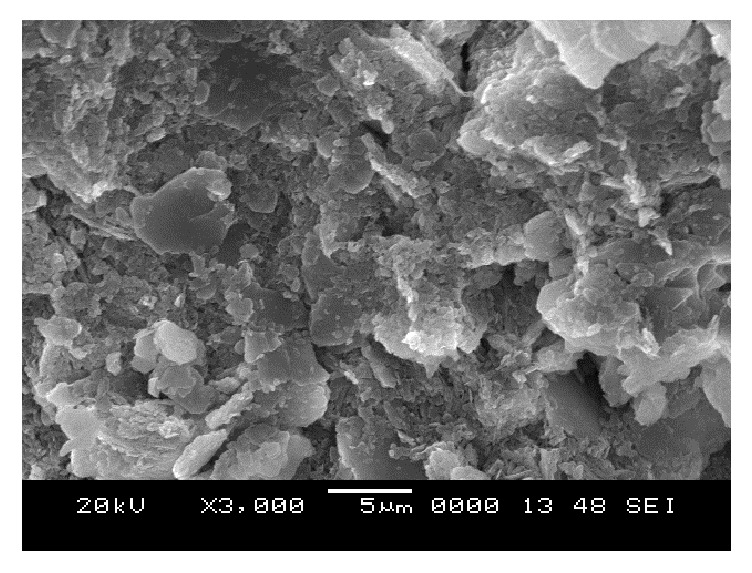
SEM image of soil-cement mix A2, after 6 days' curing.

**Figure 32:**
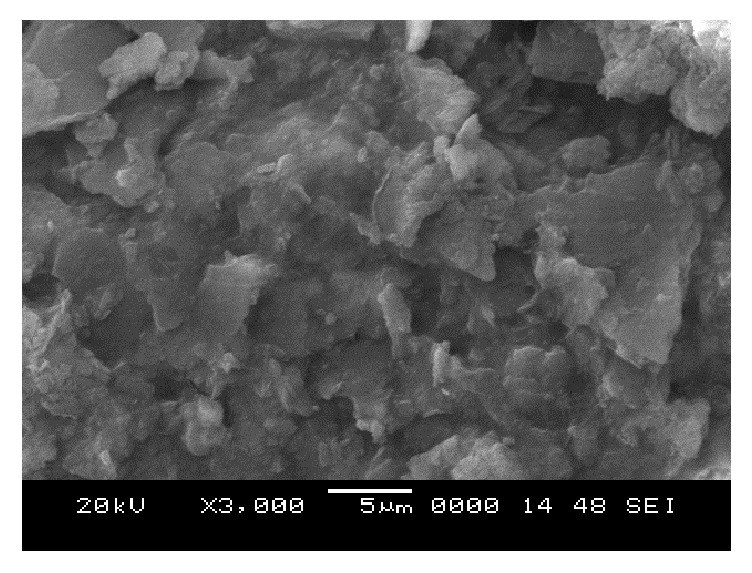
SEM image of soil-cement mix A2, after 7 days' curing.

From SEM photos for sample A2 at different curing periods (as shown in Figures 25–32), it is observed that the pore spaces or the conductive path rapidly decreases with curing time. The structure becomes more clustered with lesser voids with increase of curing time. This is because of the formation of hydration products which fills in the pore spaces and develops the bond strength and increases the resistivity with curing time. Similarly for all the other samples A4, A6, B2, B4, B6, C2, C4, C6, D2, D4, and D6 also, the micro structure becomes more dense and clustered with increase in curing time.

### 6.6. Resistivity with Unconfined Compressive Strength (UCS)

**Figure 33:**
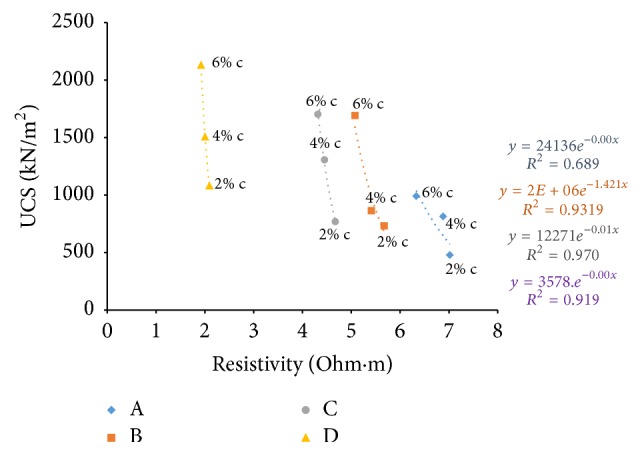
7-day UCS versus resistivity (at freshly prepared state).

**Figure 34:**
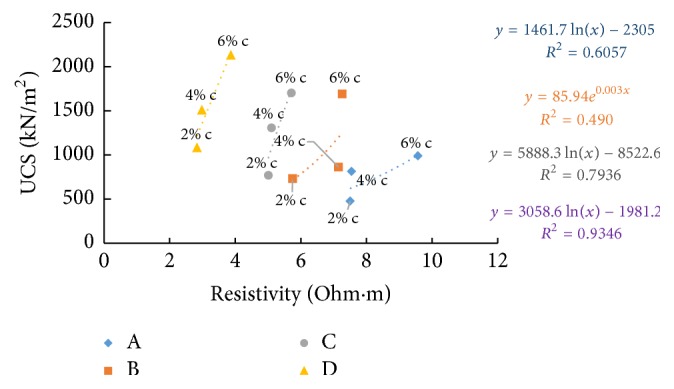
7-day UCS versus resistivity (after 1-hour curing).

**Figure 35:**
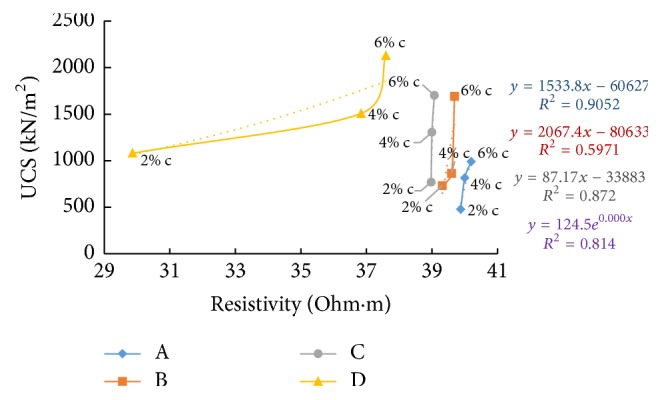
7-day UCS versus resistivity (after 7 days' curing).

Figures 33–35 show resistivity variation at different times such as in the freshly prepared state, after one hour of curing, and after seven days of curing with 7th day. With the increase in percentage of cement and river sand added, UCS is found to increase as particles become more clustered and get bonded by the cementing action and the sand particles which replace the finer particles of soil take up more load. The difference in controlling parameters of the electrical resistivity and the compressive strength such as ion concentration in pore fluid and surface charges of the soil particles, which are factors affecting ER but not UCS, was suggested as the reason behind the nonlinear relationship between electrical resistivity and unconfined compressive strength of soil-cement by Zhang et al. [4].

From Figure 33, an inverse relation is observed, between UCS (after 7 days' curing) and resistivity (of freshly prepared samples) when the cement content is varied. At freshly prepared state, as the cement content increases electrical resistivity decreases due to high electrical conduction exhibited by the ions released due to chemical reactions by cement and water, which gradually slows down with time. At the same time, electrical resistivity after 1-hour curing and 7 days' curing shows a direct relation with the unconfined compressive strength of soil-cement with increase in cement content (Figures 34 and 35).

Resistivity also follows the same trend as UCS with time of curing and increase in cement content; the samples with higher cement showed higher resistivity, since more hydration products formed fill the pore spaces and create a highly tortuous structure, bringing down the electrical conduction.

Multiple regression analysis carried out derived generalised equations which predicts the 7-day UCS of cement stabilized soil, by using the cement content (%) and the resistivity (Ohm·m) measured at freshly prepared state and also after 1-hour curing period. The regression coefficients are 0.9 and 0.95 for (3) and (4), respectively. The equations are as follows.

(3) UCS kN/m2=197.3xc %−164.3xρ0 Ohm·m+1147.7,

where *c* is the cement content and *ρ*_0_ is the electrical resistivity at freshly prepared state.

(4) UCS kN/m2=269.3xc %−160.6xρ1 Ohm·m+1035894,

where *c* is the cement content and *ρ*_1_ is the electrical resistivity measured after 1-hour curing.

### 6.7. Resistivity with Cohesion

**Figure 36:**
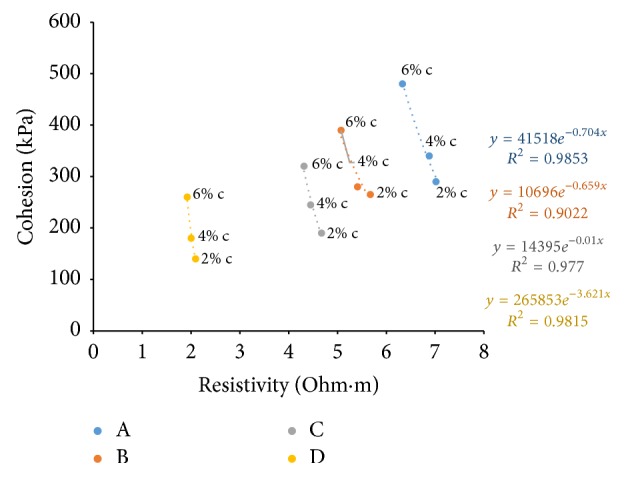
7-day cohesion with resistivity (freshly prepared).

**Figure 37:**
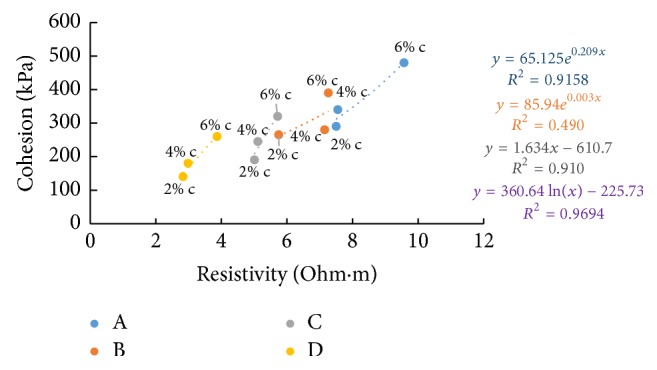
7-day cohesion with resistivity (after 1-hour curing).

**Figure 38:**
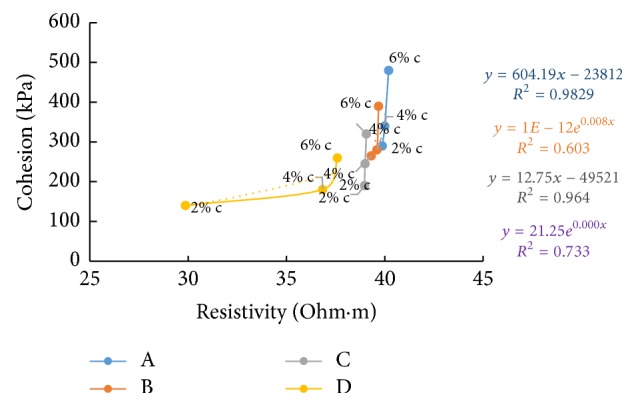
7-day cohesion with resistivity (after 7 days' curing).

Figures 36–38 show the variation of cohesion with electrical resistivity measurements in the freshly prepared state and after curing periods of one hour and seven days at different percentages of cement content for the soil samples. Cohesion after seven days' curing was found by triaxial testing. With increase of cement content, more hydration products are formed and more binding results in increase in the value of cohesion. But in the freshly prepared state, more ion concentrations result in lesser values for resistivity as the cement content increases and hence shows an inverse relation with cohesion in this state. But a direct relation is seen after curing since pore water gets used up for hydration resulting in more air in the voids which increases resistance.

### 6.8. Resistivity with Angle of Internal Friction

**Figure 39:**
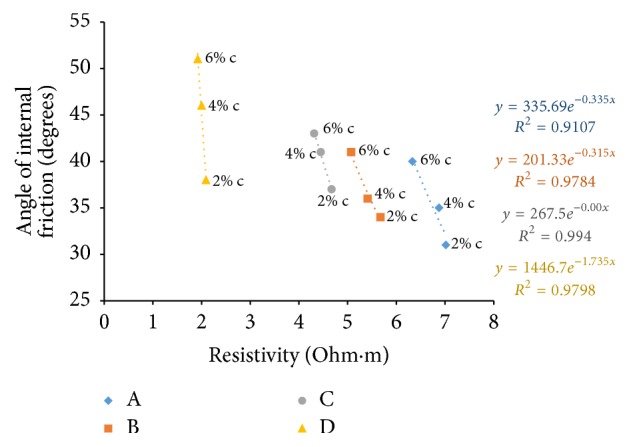
7-day angle of internal friction with resistivity (freshly prepared).

**Figure 40:**
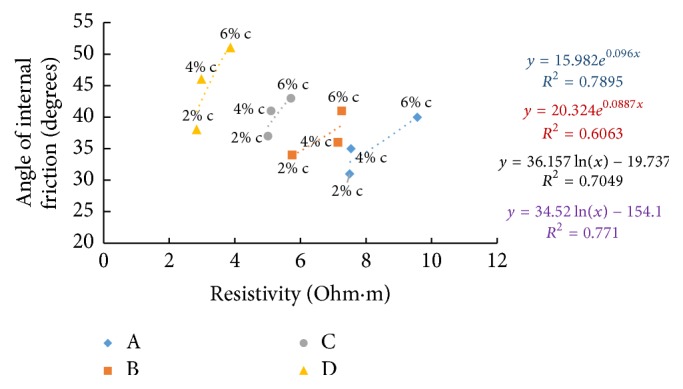
7-day angle of internal friction with resistivity (after 1-hour curing).

**Figure 41:**
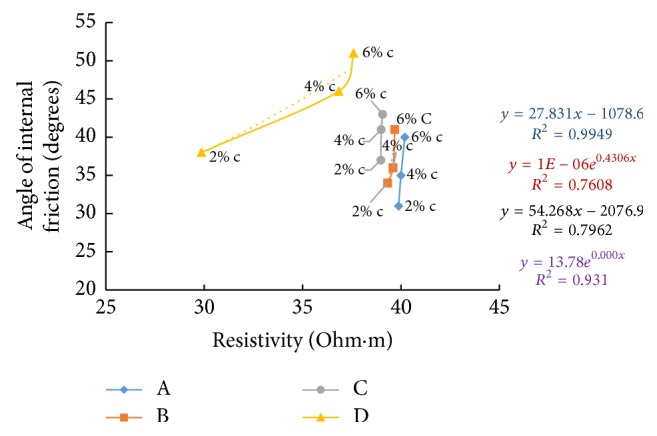
7-day angle of internal friction with resistivity (after 7 days' curing).

The shear strength parameter, angle of internal friction, was found by triaxial testing on samples after a curing period of seven days. Figures 39–41 show the variation of electrical resistivity with angle of internal friction. As cement content increases, angle of friction is found to increase which results in an inverse relation with resistivity in the freshly prepared state and direct relation in all other curing periods when plotted for all the soil-cement samples.

### 6.9. Resistivity with Split Tensile Strength

**Figure 42:**
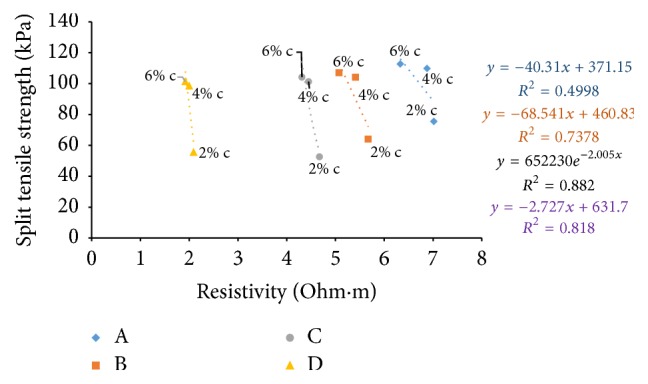
7-day split tensile strength with resistivity (freshly prepared).

**Figure 43:**
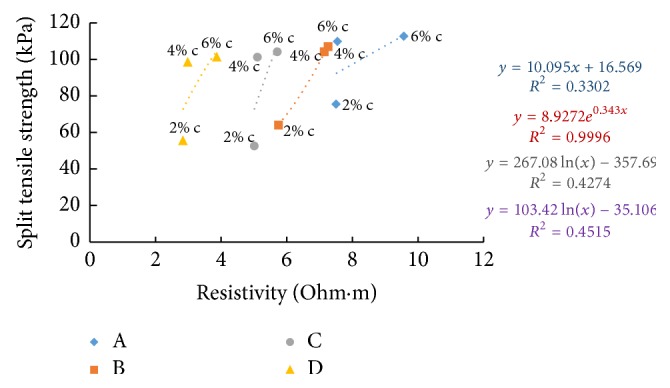
7-day split tensile strength with resistivity (after 1-hour curing).

**Figure 44:**
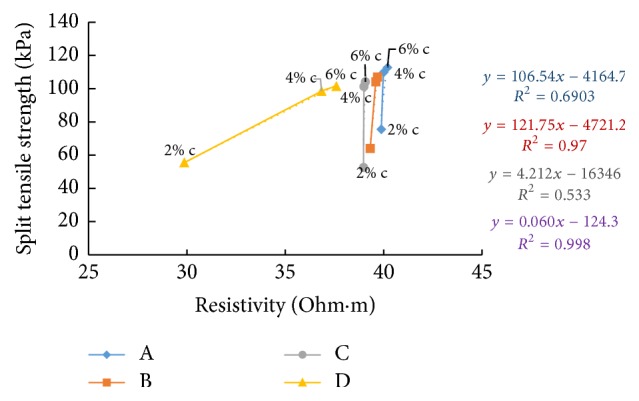
7-day split tensile strength with resistivity (after 7 days' curing).

Split tensile strength after seven days' curing time and resistivity in the freshly prepared state and after curing periods of one hour and seven days is correlated in Figures 42–44. When cement content increases, the binding increases and hence the samples with higher cement content can take up more load when tested resulting in increase of split tensile strength value. For all the soil samples, when the cement content is varied, resistivity shows indirect relation with split tensile strength for fresh samples and direct relation for cured samples.

## 7. Conclusions

In this study, electrical resistivity measurement of freshly prepared uncured and cured soil-cement materials is done and the correlation between the factors controlling the performance of soil-cement and electrical resistivity are studied. By the time an unconfined compressive strength test can be performed, to check the quality of the soil-cement, the material will be hardened in the field and if it does not meet strength and performance criteria, the material will have to be removed, collapsed, and remixed with additional cement which is a very time and cost consuming task. At this phase, electrical measurements of soil-cement/lime material save a great deal of expense and time by predicting the strength properties without hardening of the material. Equations developed in this study, by multiple regression analysis, predict the unconfined compressive strength of the soil-cement samples, at the freshly prepared state or after 1-hour curing. If the strength requirement is not met, it could be remixed with additional cement at the fresh state itself and reused.

## Figures and Tables

**Table 1:** Compaction and strength characteristics of the soil cement samples.

Parameter	Soil-cement samples															
	A0	A2	A4	A6	B0	B2	B4	B6	C0	C2	C4	C6	D0	D2	D4	D6
*γ*_*d*max_, kN/m^3^ (LC)	14.5	14.6	14.8	14.6	15.6	15.6	15.0	15.4	15.9	15.7	15.6	15.6	16.4	16.6	16.7	16.6
OMC (%) (LC)	28.0	28.3	26.8	25.6	25.0	22.4	25.2	24	23.2	22.8	23.4	23.2	21.0	20.6	18.8	20.4
*γ*_*d*max_, (kN/m^3^) (HC)	14.9	16.4	16.4	16.5	15.9	17.3	17.3	17.7	16.4	18.3	18.4	18.2	17.5	18.8	18.4	18.4
OMC (%) (HC)	27.0	22.1	21.9	21.6	22.0	19.0	19.0	17.2	21.0	14.4	16	16.5	18.0	14.9	15.4	16
7-day UCS (kPa) (LC)	130.8	478.8	814.8	991.7	155.6	733	863.6	1691.8	162.2	769.3	1307.3	1703.5	289.2	1080.8	1507.5	2130.2
C (kPa) (LC)	23.5	290.0	340.0	480.0	21.0	265.0	280.0	390.0	19.0	190.0	245.0	320.0	18.5	140.0	180.0	260.0
Φ (degrees) (LC)	20.0	31.0	35.0	40.0	25.0	34.0	36.0	41.0	31.0	37.0	41.0	43.0	31.0	38.0	46.0	51.0
STS (kPa) (LC)	—	75.52	109.9	112.8	—	64.1	104.2	107.03	—	52.6	101.3	104.2	—	55.5	98.4	101.3

*Note.γ*_*d*max_: max dry density, OMC: optimum moisture content, LC: light compaction, HC: heavy compaction, UCS: unconfined compressive strength, C: cohesion, Φ: angle of internal friction, and STS: split tensile strength.
